# An ectopic adreocortical adenoma of the renal sinus: a case report and literature review

**DOI:** 10.1186/s12894-016-0123-0

**Published:** 2016-01-16

**Authors:** Jiexiu Zhang, Bianjiang Liu, Ninghong Song, Qiang Lv, Zenjun Wang, Ming Gu

**Affiliations:** Department of Urology, The First Affiliated Hospital of Nanjing Medical University, Nanjing, 210029 China

**Keywords:** Ectopic, Adrenal tumor, CTA, Laparoscopy

## Abstract

**Background:**

Ectopic adrenal tumors are very rare, especially in the renal sinus in adults. An unusual case of ectopic adrenal cortical adenoma in the right renal sinus is reported here.

**Case presentation:**

This patient was a 37-year-old woman. She was admitted to our hospital for hypertension and bilateral limb weakness. Computed tomography (CT) revealed a mass in right renal sinus. It was initially considered a tumor of the renal pelvis. Further computed tomographic angiography (CTA) showed the mass to be located outside the renal pelvis. After adequate preoperative preparation (blood pressure control and serum potassium supplement), the patient underwent laparoscopic resection of retroperitoneal tumor. During the procedure, a soft tissue tumor 3.4*3.0 cm^2^ in size with a golden color was found in the right renal sinus. The final immunohistochemistry examination showed an ectopic adreocortical adenoma.

**Conclusion:**

Ectopic adrenal tumors are rare in the renal sinus and difficult to diagnose and treat. Large and functional tumors should be treated with complete resection. The procedure is sometimes difficult for tumors located deep in the renal sinus. The decision to perform an open or minimally invasive surgery should be made according to the surgeon’s experience.

## Background

The adrenals are derived from primordial mesenchyme in the wall of the dorsal coelom adjacent to the dorsal mesentery and urogenital structures [1]. Most ectopic adrenocortical tissues exist along the path of embryonic migration within the urogenital tract [1]. The most common sites of ectopic adrenocortical neoplasm include the celiac axis (32 %), broad ligament (23 %), adenexa of the testis (7.5 %), and spermatic cord (3–8 %) [2]. Adrenal neoplasm adjacent to the renal sinus is very rare. The present work reports an unusual case of ectopic adrenocortical adenoma in the right renal sinus.

## Case presentation

**Fig. 1:**
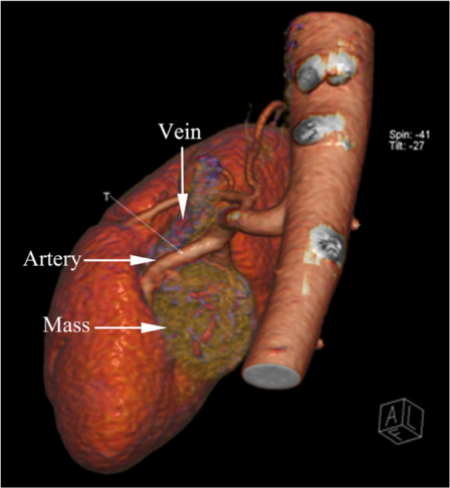
The preoperative computed tomographic angiography (CTA) image of the ectopic adrenal tumor

The patient was a 37-year-old woman. She was admitted to our hospital for hypertension and bilateral limb weakness on December 10, 2014. She had begun to take oral antihypertensive drugs 2 years earlier. The blood pressure was well controlled initially. Since October, 2014, she had felt bilateral limb weakness. Biochemical tests in a local hospital showed a significant decrease in serum potassium levels. She came to our hospital’s endocrinology department for further diagnosis and treatment. During hospitalization, her fasting blood glucose, cortisol, and aldosterone were found to be higher than normal levels. Catecholamine levels were normal. Serum potassium levels were significantly lower than normal level. Physical examination was normal. No symptoms of virilization were observed. Computed tomography (CT) of the adrenal area showed a right retroperitoneal mass (about 3.4*3.0 cm^2^) compressing the renal pelvis. The native adrenals appeared normal on the imaging. Then she was transferred to our department. Computed tomographic angiography (CTA) showed the mass to be located in the right renal sinus. It had clear margins and obvious enhancement. The mass was about 2.99*2.88*2.23 cm^3^ in size. The right renal vessel, renal pelvis and ureter were compressed by the mass (Fig. 1). A preliminary diagnosis of ectopic adrenal cortical adenoma was considered.

**Fig. 2:**
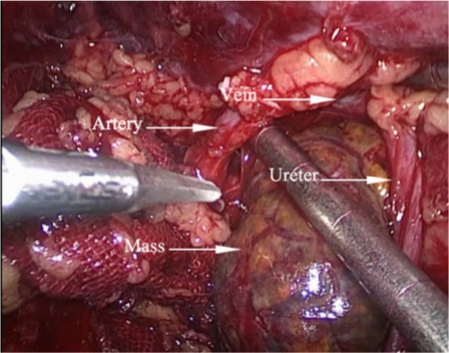
The real time image of the ectopic adrenal tumor during the operation

**Fig. 3:**
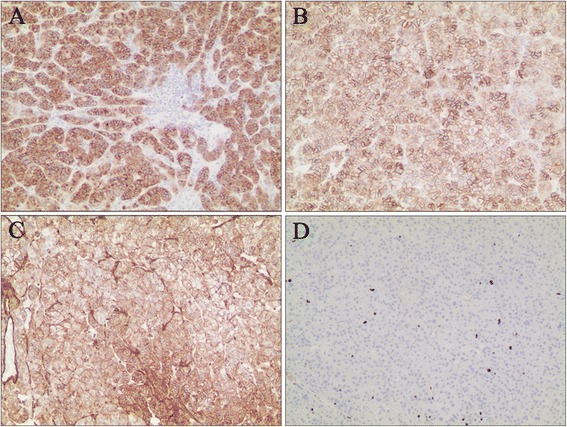
Immunohistochemistry showed positive staining for synaptophysin (**a**), CD56 (**b**), vimentin (**c**), and Ki-67(2 %) (**d**). Magnification was × 100

**Fig. 4:**
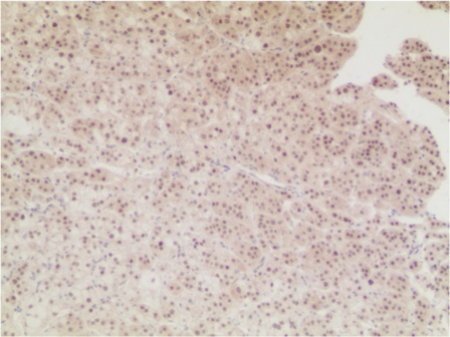
Immunohistochemistry showed ± staining for pax-8. Magnification was × 100

After adequate blood pressure control and serum potassium supplement, the patient underwent the laparoscopic resection of retroperitoneal tumor. During the operation, a mass 3.4*3.0 cm^2^ in size of medium density was found in the right renal sinus. It was tightly surrounded by the renal artery, renal vein, renal pelvis, and ureter (Fig. 2). The tumor was completely resected without conversion to open surgery. No complications were observed. The operation took about 100 min. Blood loss was about 90 ml. The blood pressure and heart rate remained stable during operation. Peri-operative vital signs and related biochemical indicators were closely monitored and remained normal. The Foley catheter was removed 1 day after the operation and the drainage tube was removed 3 days after the operation. The postoperative hospital stay lasted 4 days. Pathologic examination showed ectopic adrenal tissue with adrenocortical adenoma. Further immunohistochemistry showed the tissue to be positive for synaptophysin (Fig. 3a), CD56 (Fig. 3b), vimentin (Fig. 3c), Ki-67(2 %) (Fig. 3d), calretinin, and inhibin-α and negative for chromogranin A, CD117, CD10, CK7, EMA, CK-pan, and melan-A. However, the concentration of pax-8 was ± (Fig. 4). Using the results and HE staining, the tumor was diagnosed as an ectopic adreocortical adenoma of the right renal sinus. The patient was followed up for 1 month after the operation. Her blood pressure was normal. The bilateral limb weakness was significantly reduced. Abdominal CT showed native adrenals and no obvious mass in the renal sinus.

## Conclusions

Ectopic adrenal tissue is estimated to occur in about 1 % of the adult population and up to 50 % of neonates [3]. It regresses usually in early infancy. The adrenal cortex is derived from the coelomic mesoderm of the urogenital ridge at the 5th week of gestational age and is separated at the 8th week. Ectopic adrenal tissue occurs when a fragment of the primitive adrenal gland sheds off during development. It may come to rest in any visceral organs, especially the kidneys, liver, and gonads. Rarer sites include the lung, spinal region, stomach, and brain [1, 4]. Ectopic adrenal tissue contains cortex and medulla if the breaking event occurs after the migration of neural crest tissue into the cortex. Otherwise, only cortex exists in ectopic adrenal tissue.

Most ectopic adrenal tissue has no obvious physiological function and causes no clinical symptoms. Few ectopic adrenal tumors can produce hormones. However, this can lead to physical changes such as hypertension, feebleness, crinosity, and palpitations. The reported case involved abnormal blood glucose, cortisol, and aldsterone levels, and obvious clinical symptoms. It should be considered a functional ectopic adrenal tumor. CT is sensitive enough to indicate the locations of ectopic adrenal masses. However, it can be difficult to determine whether a mass is inside or outside of the renal pelvis if it is adjacent to renal sinus. In these cases, CTA can help determine the exact location. It is especially effective in the description of the relationship between mass and surrounding vessels, which is very important for this type of surgery.

Differentiating between benign and malignant ectopic adrenal tumor is a challenge. Routine pathological examinations are sometimes not always enough. Tumor weight and size, hormone levels, signs of vascular invasion, and high mitotic index are useful morphologic indicators to evaluate the carcinogenic potential. Large tumors (more than 100 g in weight or 5.0 cm in diameter), invasion of surrounding tissues, and presence of metastasis, are indicators of malignancy [5, 6]. In addition, malignant tumor presents more commonly a mixed Cushing-virilization syndrome. The tumor in this report was much smaller than 100 g in weight and 3.4 cm in diameter. Only abnormal cortisol and aldsterone levels were detected. The tumor was completely resected from the renal sinus with an intact capsule. No invasion of surrounding tissues was detected. All signs indicated the benign tendency of this mass. Recently, some molecular markers have been used to differentiate adrenal carcinoma from benign tumors [7]. These new markers may help to distinguish the nature of tumor in the future.

The treatment of ectopic adrenal tumors includes conservative therapy and radical resection (open or minimally invasive surgery). If the tumor is small or nonfunctioning, watchful waiting is enough. Otherwise, the tumor should be resected although evincing no malignant tendency. In our study, the large tumor size (3.4 cm in diameter) and obvious clinical symptoms can serve at surgical indications. However, the procedure is complicated and difficult because tumors in the renal sinus are surrounded by the renal artery, renal vein, and ureter. Common complications include uncontrolled bleeding and side injury of renal pelvis or ureter. The choice of a surgical approach should depend on the surgeon’s experience.

In summary, ectopic adrenal tumors of the renal sinus are rare and difficult to diagnose and treat. For large or functional tumors, complete resection should be performed. The procedure can be difficult if the tumor is located deep in the renal sinus. The decision to perform an open or minimally invasive surgery should be made according to the surgeon’s experience.

## Consent

Written informed consent was obtained from the patient for publication of this Case report and any accompanying images. A copy of the written consent is available for review by the Editor of this journal.

## Abbreviations

CT: Computed tomography
CTA: Computed tomographic angiography

## References

[1] Ren PT, Fu H, He XW (2013). Ectopic adrenal cortical adenoma in the gastric wall: case report. World J Gastroenterol.

[2] Makino K, Kojima R, Nakamura H, Morioka M, Iyama K, Shigematsu K (2010). Ectopic adrenal cortical adenoma in the spinal region: case report and review of the literature. Brain Tumor Pathol.

[3] Souverijns G, Peene P, Keuleers H, Vanbockrijck M (2000). Ectopic localisation of adrenal cortex. Eur Radiol.

[4] Ye H, Yoon GS, Epstein JI (2009). Intrarenal ectopic adrenal tissue and renal-adrenal fusion: a report of nine cases. Mod Pathol.

[5] Lee PDK, Winter RJ, Green OC (1985). Virilizing adrenocortical tumors in childhood: eight cases and review of the literature. Pediatrics.

[6] Wolthers OD, Cameron FJ, Scheimberg I, Honour JW, Hindmarsh PC, Savage MO (1999). Androgen secreting adrenocortical tumors. Arch Dis Child.

[7] Choukair D, Beuschlein F, Zwermann O, Wudy SA, Haufe S, Holland-Cunz S (2013). Virilization of a young girl caused by concomitant ectopic and intra-adrenal adenomas of the adrenal cortex. Horm Res Paediatr.

